# One stone two birds: anti-inflammatory bronchodilators as a potential pharmacological strategy for COVID-19

**DOI:** 10.3389/fphar.2023.1185076

**Published:** 2023-05-04

**Authors:** Yuanyuan Yu, Bangjiang Fang, Xiao-Dong Yang, Yuejuan Zheng

**Affiliations:** ^1^ The Research Center for Traditional Chinese Medicine, Shanghai Institute of Infectious Diseases and Biosecurity, Shanghai University of Traditional Chinese Medicine, Shanghai, China; ^2^ Center for Traditional Chinese Medicine and Immunology Research, School of Basic Medical Sciences, Shanghai University of Traditional Chinese Medicine, Shanghai, China; ^3^ Department of Emergency, LongHua Hospital, Shanghai University of Traditional Chinese Medicine, Shanghai, China

**Keywords:** bronchodilators, COVID-19, respiratory, anti-inflammatory, bronchodilation

## Abstract

The ongoing Coronavirus disease 2019 (COVID-19) pandemic caused by severe acute respiratory syndrome coronavirus 2 (SARS-CoV-2) has imposed a huge threat to public health across the world. While vaccinations are essential for reducing virus transmission and attenuating disease severity, the nature of high mutation rate of SARS-CoV-2 renders vaccines less effective, urging quick development of effective therapies for COVID-19 disease. However, developing novel drugs remains extremely challenging due to the lengthy process and high cost. Alternatively, repurposing of existing drugs on the market represents a rapid and safe strategy for combating COVID-19 pandemic. Bronchodilators are first line drugs for inflammatory lung diseases, such as asthma and chronic obstructive pulmonary disease (COPD). Compared to other anti-inflammatory drugs repurposed for COVID-19, bronchodilators are unique in that they have both anti-inflammatory and bronchodilating properties. Whether the dual properties of bronchodilators empower them greater potential to be repurposed for COVID-19 is worth exploring. In fact, clinical and preclinical studies have recently emerged to investigate the benefits of bronchodilators such assalbutamol, formoterol and theophylline in treating COVID-19, and many of them have shown encouraging efficacy on attenuating disease severity of pneumonia and other associated symptoms. To comprehensively understand the latest progress on COVID-19 intervention with bronchodilators, this review will summarize recent findings in this area and highlight the promising clinical benefits and possible adverse effects of bronchodilators as therapeutic options for COVID-19 with a focus on β2 receptor agonists, anticholinergic drugs and theophylline.

## 1 Introduction of SARS-CoV-2

The emergence of SARS-CoV-2 was reported in December 2019 and could cause COVID-19. It gained its name due to its similarity to SARS-CoV given by the International Committee on Taxonomy of Viruses (ICTV) (Coronaviridae Study Group of the International Committee on Taxonomy of V, 2020). Externally, SARS-CoV-2 are usually spherical particles with a diameter of approximately 60–220 nanometers. The SARS-CoV-2 particle, like other coronaviruses, has a number of neatly arranged spikes on its surface that look very much like one crown. The SARS-CoV-2 particles are made up of five components: single-stranded RNA and four proteins. The outermost layer of the particle contains receptor-binding spike glycoprotein (S protein), and below the spike is the viral envelope composed of envelope protein (E protein) and membrane protein (M protein) (Schoeman and Fielding, 2019; Wang et al., 2020a; Phan, 2020). Angiotensin converting enzyme 2 (ACE2) is a membrane receptor commonly expressed on epithelial cells in various organs of the human body, including lungs, digestive system, hearts, arteries and kidneys. The main function of S protein of SARS-CoV-2 is to bind to ACE2 on the surface of host cells, allowing the coronaviral particles to fuse into the cell membrane and its entry, releasing viral RNA strand to replicate themselves for next generations. The expression of ACE2 is increased with age and is relatively high in patients with cardiovascular diseases (Gheblawi et al., 2020; Xia et al., 2020; Yan et al., 2020).

The symptoms of COVID-19 vary from asymptomatic, mild, moderate, to severe and critical. Severely and critically ill COVID-19 patients have a high risk of acute respiratory distress syndrome (ARDS), infiltrating pneumonia, sepsis, septic shock, and even death (Chen and Li, 2020). Autopsy results of COVID-19 patients showed that there were many sticky sputa in bronchi and bronchioles (Wang et al., 2020b). As of 6 April 2023, 762 million people have been infected and 6.89 million have died worldwide (WHO, 2023).

## 2 Immunopathologic character of COVID-19

It has been demonstrated *in vitro* and *in vivo* that SARS-CoV-2 can infect the lung and multiple organs (To et al., 2021). Autopsy studies elucidated that the most prominent pathological characteristics of COVID-19 are diffuse alveolar damage, the formation of hyaline membrane, and pulmonary microemboli. It has been proposed that the hyaline membrane is caused by an increase in vascular permeability (referred to as the “bradykinin storm”) and an accumulation of hyaluronic acid in the alveoli, resulting in the leaking of a large amount of fluid into alveoli (Garvin et al., 2020). And thus oxygen exchange is decreased and hypoxemia occurs frequently. Many studies have discovered that pathogen associated molecular patterns (PAMPs) derived from invading SARS-CoV-2 can be recognized by pattern recognition receptors (PRRs) expressed on host cells, which in turn activate inflammatory signaling pathways to induce NF-κB- and IRF3/7-dependent transcription of cytokines and inflammatory mediators (Bortolotti et al., 2021). Virus-induced inflammatory response is essential for the defense against viral infections, but can cause fairly detrimental consequences when dysregulated. Plenty of evidences indicated that in COVID-19 patients, particularly those admitted to an intensive care unit (ICU) (McDonald et al., 1979), have extremely high levels of inflammatory cytokines and chemokines like IL-1β, IL-2, IL-6, IFN-γ, TNF-α, IL-10, IP-10, GM-CSF and MCP-1, which are correlated with the severity of the disease (Zanza et al., 2022). It is therefore suggested that cytokine storm caused by over-activated inflammatory cascade is the key pathogenic factor of COVID-19. In addition to causing direct tissue damages, cytokine storm are often accompanied by massive infiltration of immune cells, dominated by neutrophils and macrophages, which further fuel up the production of cytokines, resulting in severe bilateral pneumonia, ARDS, and even multiple organ failure (Domingo et al., 2020).

As central controllers of proinflammatory cytokine IL-1β maturation and secretion, inflammasomes, primarily the NLRP3 inflammasome, can also be activated by SARS-CoV-2-derived PAMPs and damage-associated molecular patterns (DAMPs) released from virus-infected host cells, eliciting IL-1β- and pyroptotic cell death-dependent systemic inflammation (Pan et al., 2021; Vora et al., 2021). Clinical and animal model studies have demonstrated that the overactivation of NLRP3 inflammasome plays a critical role in the pathogenesis of severe COVID-19 disease (Vora et al., 2021; Sefik et al., 2022).

The immunopathology of COVID-19 strongly implies that targeting the overwhelmed inflammatory response and thus reversing respiratory dysfunction could be a viable strategy to reduce its morbidity and mortality.

**FIGURE 1:**
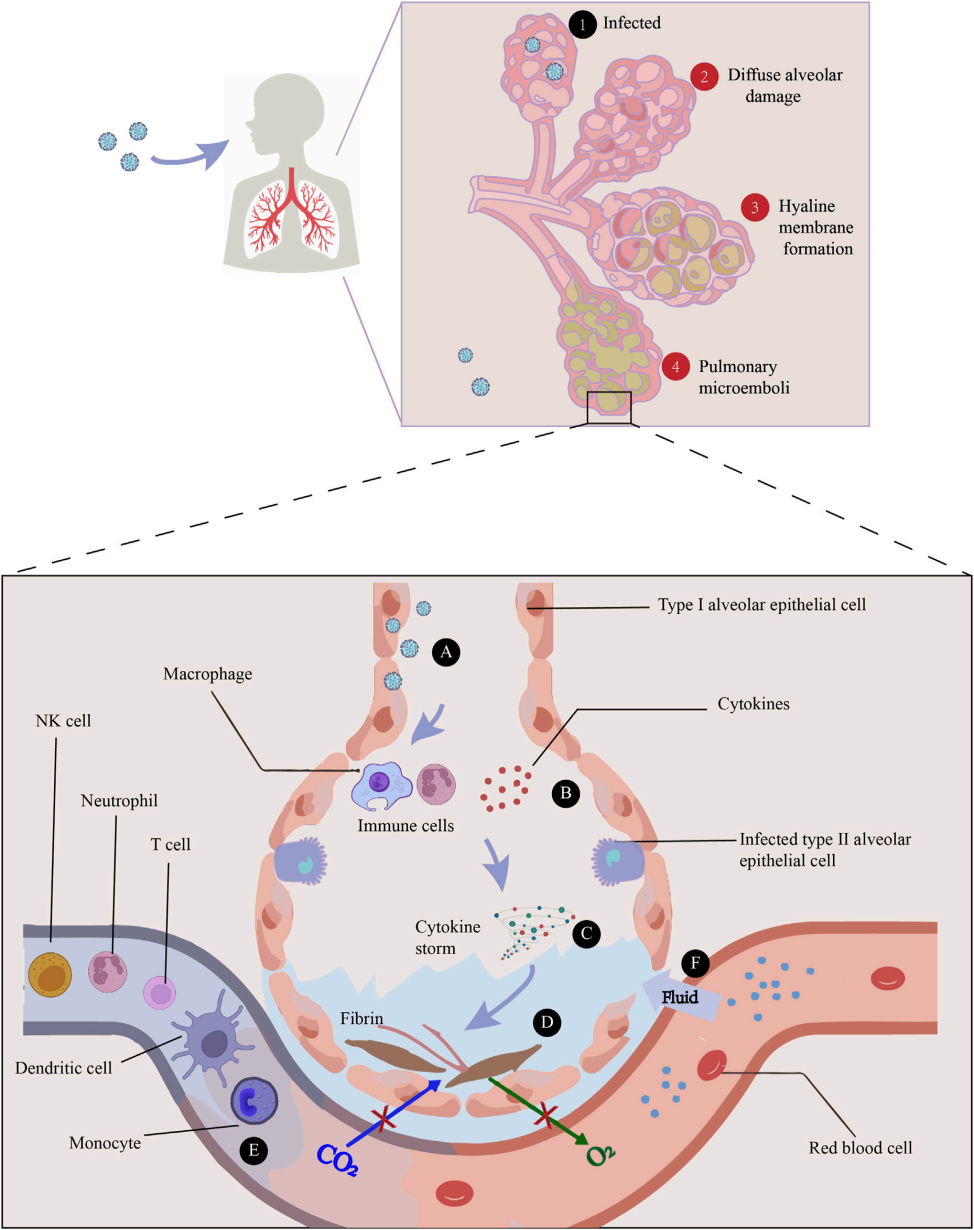
The main pathogenesis of COVID-19 is immune dysfunction, manifested by a series of sequential events: ① Infection of the alveoli by viruses, ②Diffuse alveolar damage caused by SARS-CoV-2, ③Hyaline membrane resulted from an increase in vascular permeability (referred to as the “bradykinin storm”) and an accumulation of hyaluronic acid in the alveolar space, and ④Pulmonary microemboli in the lung. A) The infection of lung cells can cause a rapid recall of infiltration of inflammatory cells. B) Immune cells, such as macrophages, recognize the virus and release cytokines. C) Cytokine storm is the key pathogenic factor of COVID-19, showing increased levels of inflammatory cytokines like IL-1β, IL-2, IL-6, IFN-γ, TNF-α, IL-10, IP-10, GM-CSF and MCP-1. D) Damage can happen through the formation of fibrin and pulmonary fibrosis, thus impairing gas exchange. E) In comparison with healthy donors, acute COVID-19 patients have significantly fewer dendritic cells, T cells, NK cells, and monocytes in their peripheral blood. F) Increased permeability of blood vessels allow fluid to seep into the air sacs, increasing the dysfunction of ventilation.

The first line of defense against infection is innate immunity. However, SARS-CoV-2 may be able to evade innate immunity by antagonistically interacting with host interferons (Yuen et al., 2020). In addition, in comparison with healthy donors, acute patients had significantly fewer dendritic cells, T cells, NK cells, and monocytes in their peripheral blood (Zhou et al., 2020). As for the humoral adaptive immune response, most recovered patients develop antibodies (IgA, IgG and IgM) specifically directed against SARS-CoV-2, including both S and N proteins, as well as other nonstructural proteins (Jiang et al., 2020). In terms of cellular adaptive immunity, SARS-CoV-2 causes T-cell lymphopenia and impaired functional capacity of both CD4^+^ and CD8^+^ T cells during the acute phase. The number of CD4^+^ and CD8^+^ T cells is reduced in both mild and severe diseases, but in severe cases it is particularly lower (De Biasi et al., 2020) (Figure 1).

## 3 Drug repurposing to meet challenges for COVID-19 treatment

To slow down the spread of SARS-CoV-2, non-pharmaceutical measures like physical separation, proper mask use, teleworking, isolations, and quarantines have been implemented throughout the pandemic. The above-mentioned efforts at compliance not only impede economic and social development, but also often cause undesirable health concerns, such as depressions and mental health repercussions (Phan, 2020). Many efforts have been made to prevent and treat COVID-19 worldwide. First of all, a variety of vaccines against SARS-CoV-2 were successfully developed soon after the outbreak of the pandemic, and currently at least nine vaccines are available at the market from Pfizer‐BioNTech, Gamaleya, Moderna, Oxford‐AstraZeneca, Novavax, Bharat Biotech, Sinopharm, Johnson & Johnson and Sinovac companies (Hadj Hassine, 2022). The protective efficiencies of various vaccines have been reported ranging from 60% to 94% (Tregoning et al., 2021) and global vaccination had substantially reduced the morbidity of severe COVID-19 cases. Unfortunately, the emergence of SARS-CoV-2 variants, such as Alpha, Beta, Gamma, Delta and Omicron strains keep increasing the risk for vaccine escape and drug resistance (Brüssow, 2021). Secondly, some high-risk populations, e.g., the elderly, the immunodeficient and those with co-morbidities such as obesity, diabetes mellitus, and cardiovascular diseases, are not suitable to take vaccines or lack proper response to vaccination and therefore remain vulnerable to SARS-CoV-2 infection. Thus, the development of effective and alternative therapies would be crucial to cope with COVID-19 pandemic globally.

Compared to *de novo* drug discovery process that often takes a long time, high cost and unpredictable risk, repurposing of currently available drugs offers alternative approaches to rational drug design for COVID-19 (Wang, 2020). A large number of existing drugs have been and are being repurposed for the treatment of COVID-19. They include but are not limited to the following five types of drugs: 1) anti-virus immunotherapies, such as molnupiravir and remdesivir, 2) Corticosteroids, such as dexamethasone, 3) anti-cytokine antibodies, such as anti-IL-6 antibodies (tocilizumab, sarilumab), anti-IL-1 antibody (anakinra), anti-TNF antibody (adalimumab) and anti-GM-CSF antibody, 4) inhibitors of signaling pathways, such as JAK inhibitors (baricitinib, tofactinib) and PI3K/mTOR inhibitors, 5) Kinase inhibitors, such as tyrosine kinase inhibitors, 6) Inhibitors of local pulmonary edema: kallikrein–kinin system, such as icatibant, and 7) antimalarials, such as chloroquine and hydroxychloroquine, etc. Quite a few of such repurposed drugs have displayed beneficial effect on reducing disease severity and mortality, accompanied by controllable adverse reactions (van de Veerdonk et al., 2022, Giamarellos-Bourboulis, Pickkers, Derde, Leavis, van Crevel, et al.; Saravolatz et al., 2023; Aiello et al., 2022).

Bronchodilators are good and unique repurposed drug candidates for COVID-19 because of their bronchodilating and anti-inflammatory bioactivities, which are supposed to be beneficial to prevent the deterioration of disease. This review aims to summarize the applications of bronchodilators in the treatment of COVID-19 and discuss the underlying mechanisms focusing on their immunomodulatory roles.

## 4 Bronchodilators as potential therapeutic options for COVID-19

### 4.1 Bronchodilators are the mainstay for the treatment of pulmonary disease

Rapid airflow, turbulent airflow and reduced airway diameter can increase airway resistance and affect pulmonary ventilation. Bronchodilators can relax the bronchial smooth muscles and have excellent dilation effect on small airways. Due to the above functions, bronchodilators can significantly improve the pulmonary function of patients with bronchial asthma, chronic obstructive pulmonary disease (COPD), bronchospasm and other airflow restricted lung diseases (Matera et al., 2020). The frequently-used bronchodilators include β2 receptor agonists, anticholinergic and theophylline drugs.

It is verified by a clinical trial in COPD patients that using a combination of long-acting β2-adrenoceptor agonists and long-acting muscarinic acetylcholine receptor antagonists plays a synergistic role on the relaxation of airway smooth muscles (ASM) (Calzetta et al., 2018). For the treatment of COPD patients, triple inhalers, a combination of β2-adrenergic receptor antagonists, muscarinic receptor antagonists and inhaled corticosteroids, have received clinical approval (Matera et al., 2020).

### 4.2 Therapeutic potentials of bronchodilators to treat COVID-19

During the pandemic of COVID-19, bronchodilators have been repurposed to treat COVID-19 patients and encouraging efficacy has been achieved, suggesting promising potential to be therapeutic options for COVID-19 (Elbeddini, 2020; Maniscalco et al., 2021a; O’Keefe et al., 2021).

#### 4.2.1 β2 adrenoceptor agonists

β2 adrenoceptor agonists are used to relieve asthma symptoms by stimulating β2 receptors on the surface of airway smooth muscle and mast cells, relaxing airway smooth muscle, inhibiting the release of inflammatory mediators from mast cells and basophils, reducing microvascular permeability, and increasing airway epithelial ciliary oscillation. Based on the acting duration time, they can be divided into short-acting (4–6 h of action) agonists, such as terbutaline and salbutamol, and long-acting (12 h of action) agonists, such as formoterol and salmeterol (Xing et al., 2021).
β2 adrenoceptor agonists were used to treat the patients with moderate and severe COVID-19 (Procter et al., 2020), Salbutamol metered dose inhaler (MDI) has become an important supportive option for emergent treatment of patients with moderate and severe COVID-19 (Elbeddini, 2020). The study revealed that the use of bronchodilators was beneficial for the rehabilitation from mild to severe stages of COVID-19 patients, because it induced improvement in lung function and promotes respiration (Maniscalco et al., 2021b). Salbutamol is often used to treat lower respiratory symptoms in patients with mild COVID-19 and has been reported to be one of the most effective drugs (O’Keefe et al., 2021). The study revealed that in post-COVID-19 patients, greatest benefit of salbutamol was seen in patients with asthma, followed by patients with COPD, and finally, patients without COPD (Maniscalco et al., 2021a). In addition, combined treatment with β2 adrenoceptor agonists and other drugs had an impressive protective role in COVID-19 patients. For instance, the combination of salbutamol and dornase alfa, a mucilage dissolver, reduced the incidence of mechanical ventilation or intubation for severe COVID-19 patients (Weber et al., 2020). The combination of dexamethasone with formoterol or salmeterol was proposed to improve lung function, assist symptom control, and decrease exacerbations (Hajjo et al., 2020).

One of the main therapeutic effects of β2 adrenoceptor agonists on COVID-19 could be attributable to their anti-inflammatory activities that have been discovered in non-COVID-19 settings. The infiltration and activation of neutrophils in lungs play a vital role during the immunopathologic injury of COVID-19 patients (Cesta et al., 2023). A clinical study showed that equine asthma patients treated with fluticasone/salmeterol experienced reduced neutrophilic infiltration and airway remodeling (Bullone et al., 2017). The decreased infiltration and activation of neutrophils by fluticasone/salmeterol may partially account for their therapeutic effects on COVID-19 (Cesta et al., 2023). An animal model study revealed that Salmeterol suppressed LPS-induced systemic inflammation *in vivo* by inhibiting the activation of NLRP3 inflammasome (Song et al., 2018) which was recently shown to greatly contribute to the pathology of COVID-19 in a humanized mouse model (Sefik et al., 2022). Salbutamol and terbutaline reduced cytokine production in macrophages via inhibiting ERK activation through cAMP (Keränen et al., 2017). Formoterol and indacaterol reduce the secretion of proinflammatory cytokines in neutrophils *in vitro* (Anderson et al., 2014). The activation of dendritic cell by salbutamol encourages CD4^+^ T cells to secrete IL-10 (Hervé et al., 2017). Through activating cAMP/Epac signaling, terbutaline protects pulmonary microvascular endothelial cells from LPS-induced damage (Duan et al., 2022). (R)-salbutamol significantly suppressed LPS-induced polarization of M1 macrophages and decreased the expression of M1 typical cytokines through down-regulating the metabolism of aerobic glycolysis and glycerophospholipid (Wang et al., 2020c). Further studies are needed to carry out both in clinic and animal models to decipher the pharmacological mechanism for β2 adrenoceptor agonists’ therapeutic roles on COVID-19.

#### 4.2.2 Anticholinergic drugs

Cholinergic receptor blockers can inhibit the downstream signaling pathways by competitive combination of these receptors with their ligands (acetylcholine, etc.) on bronchial smooth muscle and exhibit the role of bronchial dilation. Ipratropium is a potent anticholinergic drug with high selectivity and affinity for bronchial smooth muscle M receptors, which has a strong effect on the relaxation of smooth muscle, but a weak effect on the relaxation of respiratory glands and cardiovascular system. By blocking the smooth muscle muscarinic M3 receptor subtype, the selective anticholinergic medication trotripum causes bronchiectasis. Ipratropium refers to short- and medium-acting cholinergic receptor blockers, while tiotropium refers to the long-acting cholinergic receptor blockers.

Tiotropium was identified as a promising candidate for SARS-CoV-2 in a drug-target interaction-based prediction study of commercially available drugs using a deep learning algorithm (Beck et al., 2020). This finding was supported by a follow-up study that revealed a remarkable similarity in hyperinflammatory features between COVID-19 and COPD (Kang et al., 2020). Since tiotropium can improve pulmonary function by alleviating airway inflammation, it is reasonable to predict the effectiveness of tiotropium for COVID-19 (Kang et al., 2020). A retrospective cohort study with 612 COVID-19 patients were carried out to identify medications associated with altered disease outcomes in 2021, and the data showed that among the 42 medications tested, tiotropium was one of the very few agents associated with lower mortality, implying the positive correlation between the application of tiotropium and a good prognosis of COVID-19 patients (Oddy et al., 2021).

Studies of animal models of other pulmonary diseases, such as cadmium inhalation-, virus-, cigarette smoke-, or ambient particulate matter-induced COPD, asthma and other inflammatory disorders (Shen et al., 2014; Bucher et al., 2016; Kurai et al., 2018; Zhao et al., 2018), revealed a striking feature that tiotropium treatment reduced the infiltration of neutrophils in lungs of all above-mentioned animal models. Consistent with these animal model studies, an *in vitro* assay conducted in human bronchial epithelial cells demonstrated that tiotropium was able to inhibit LPS-induced NF-κB activation, thereby reducing the production of chemokine IL-8 (Suzaki et al., 2011), exhibiting an impeded chemotaxis for neutrophils in the infection site (Suzaki et al., 2011; Matsushima et al., 2022). Because of the important immunopathologic role of over-activated neutrophils in lungs of COVID-19 patients, tiotropium is predicted to play a similar protective role in COVID-19 (Borges et al., 2020).

#### 4.2.3 Theophylline and derivatives

Theophylline (3-methyxanthine) is an important drug used either alone or in combination with other drugs to treat COPD or asthma with a long history. As phosphodiesterase inhibitors, theophylline have a direct dilating effect on airway smooth muscles, albeit a slightly weaker than that of β2 receptor agonists. These drugs have a rapid onset of action but a relatively short duration, for example, aminophylline and sulpiride. Theophylline can also function as an adenosine receptor antagonist and responsible for its ability to relax pulmonary blood vessels (Montaño et al., 2022).

In contrast to β2 adrenoceptor agonists and anticholinergic drugs, theophylline and its derivatives have been tested to treat multiple symptoms (e.g., asthma and bronchitis) associated with COVID-19. A retrospective study to examine the potential efficacy and safety of theophylline and pentoxifylline in COVID-19 pneumonia patients demonstrated the associations of theophylline and pentoxifylline with an increase in ROX score and nominal decreases in C-reactive protein and mortality, laying the basis for further exploration of clinical benefits of these drugs for COVID-19 pneumonia (Wall et al., 2021). The underlying mechanism of their therapeutic roles in COVID-19 needs to be further explored. When used to treat COVID-19-associated sinus bradycardia caused by virus-elicited inflammation and injury, theophylline and etophylline brought heart rate within normal range in all 10 patients studied. It was therefore proposed that these drugs could be applied to treat arrhythmia in COVID-19 patients (Dahiya et al., 2022). A randomized clinical trial done in 26 adult COVID-19 patients who had olfactory dysfunction reported that theophylline at least slightly improved olfactory function compared to the placebo. Likely due to the small number of the patients enrolled, the difference was not statistically significant but would stimulate larger studies to verify the clinical benefit of theophylline on olfaction (Gupta et al., 2022). Interestingly, data from an *in silico* study showed that among 5 methylxanthines tested, theophylline exhibited the best binding affinity for all three target proteins of SARS-CoV-2, the spike protein, main protease, and nucleocapsid protein. This lays the groundwork for future studies of theophylline as an antiviral drug for COVID-19 (Rolta et al., 2022).

Continuous theophylline administration for 4 weeks at 9–11 mg/L in plasma was reported to cause a significant decrease in induced sputum inflammatory cells, primarily neutrophils, and a lowering of IL-8, myeloperoxidase, and lactoferrin in COPD patients (Culpitt et al., 2002). Theophylline (10 μM) increased TNF-α-induced *p*P2A enzymatic activity to repress IL-8 secretion in airway smooth muscle cells (Patel et al., 2016). It is worth mentioning that theophylline has been demonstrated to have anti-inflammatory effects in COPD and asthma at blood concentrations far below those required for bronchodilator action (Barnes, 2013).

Theophylline also inhibits TNF-α-induced I-κB phosphorylation and degradation and thus decreased the translocation of NF-κB into the nuclei, inhibiting the expression of target genes (e.g.,.IL-6,IL-8,TNF-α) (Table 1) (Ichiyama et al., 2001; Umeda et al., 2002; Iiboshi et al., 2007).

**TABLE 1:** Immunomodulatory effects of bronchodilators in COVID-19.

Types of drugs	Drug names	Clinical application in COVID-19	Regulation of immune responses
β2 adrenoceptor agonists	Salbutamol Terbutaline Formoterol Salmeterol	1. Salbutamol MDI has become an important support drug for the treatment of COVID-19 patients in emergency ward (Calzetta et al., 2018)	1. Reducing cytokine production by inhibiting the cAMP/ERK pathway of macrophage (Song et al., 2018)
		2. The use of bronchodilators is essential to the rehabilitation of all COVID-19 patients (Procter et al., 2020)	2. CD4^+^ T cells co-cultured with salbutamol-treated DCs decreased the secretion of TNF-α and IL-6 and increased IL-10 secretion (Anderson et al., 2014)
		3. Salbutamol is often used to treat lower respiratory symptoms in mild cases of COVID-19 (Elbeddini, 2020)	3. Inhibiting the infiltration and activation of neutrophils (Hajjo et al., 2020; Cesta et al., 2023)
		4. The combined use of salbutamol and a mucilage dissolver can reduce oxygen demand for severe COVID-19 patients requiring mechanical ventilation (Maniscalco et al., 2021b)	4. Suppressing NLRP3 Inflammasome (Bullone et al., 2017; Pan et al., 2021)
		5. The combination of dexamethasone with formoterol or salmeterol resulted in rapid relief of respiratory symptoms (Weber et al., 2020)	5. Suppressing the polarization of M1 macrophages as well as the expression of M1 macrophage’s typical cytokines, including MCP-1, IL-1β and TNF-α (Duan et al., 2022)
			6. Action on cAMP/Epac signaling (Hervé et al., 2017)
Anticholinergic drugs	Ipratropium	1. Tiotropium has been evaluated as a potential treatment for COVID-19 (Beck et al., 2020; Wang et al., 2020c)	1. Tiotropium reduced the infiltration of neutrophils in lungs (Shen et al., 2014; Bucher et al., 2016; Zhao et al., 2018; Oddy et al., 2021) 2. Suppressing the activation NF-κB, thus inhibiting IL-8 gene and protein overexpression from human airway epithelial cells and lung fibroblasts (Kurai et al., 2018)
	Tiotropium	2. Tiotropium was associated with lower mortality when applied to COVID-19 patients (Kang et al., 2020)	
Theophylline drugs	Aminophylline sulpiride	1. Theophylline and its derivatives have been tested to treat multiple symptoms (e.g.,: asthma, bronchitis, sinus bradycardia) associated with COVID-19 (Wall et al., 2021; Montaño et al., 2022)	1. The associations of theophylline and pentoxifylline with an increase in ROX score and nominal decreases in C-reactive protein and mortality (Montaño et al., 2022) 2. A significant decrease in induced sputum inflammatory cells, primarily neutrophils, and a lowering of IL-8, myeloperoxidase, and lactoferrin in COPD patients (Rolta et al., 2022) 3. Increasing TNF-α-induced *p*P2A enzymatic activity to repress IL-8 secretion in airway smooth muscle cells (Culpitt et al., 2002) 4. Inhibiting TNF-α-induced I-κB phosphorylation and degradation, and thus decreased the translocation of NF-κB into the nuclei and the expression of target genes (Ichiyama et al., 2001; Barnes, 2013; Patel et al., 2016)
		2. A study reported that theophylline at least slightly improved olfactory function compared to the placebo in COVID-19 patients who had olfactory dysfunction (Dahiya et al., 2022)	

### 4.3 Side effects of bronchodilators

The side effects of β2 agonists are mainly related to the activation of the sympathetic nervous system with the most common being shivering, tension, palpitation and muscle spasm. In severe conditions side effects can be extended to paradoxical bronchospasm, hypersensitivity reaction, hypokalemia and myocardial infarction. Patients who have been using short-acting β2 adrenergic receptor agonists for a long period of time may need an increasing dose to achieve the corresponding benefit due to the downregulation of its receptor. The main side effects of M receptor antagonists are symptoms caused by decreased vagal tone, such as dry mouth, urinary retention, tachycardia, constipation and stomach upset, etc. Since it can induce delirium in elderly patients, cautions needs to be taken when using M receptor antagonists (Nanda et al., 2020). When asthma and/or COPD exacerbations occur, patients may develop type B lactic acidosis due to excess lactate production from respiratory muscle fatigue and further elevated blood lactate levels due to the inhalation of β2 agonists to relieve symptoms of respiratory distress, which is often overlooked (Lau et al., 2013). The adverse effects of theophylline are usually dose-dependent, and when combined with phenobarbital, the clearance rate of theophylline was declined. The most common adverse effects of theophylline are gastrointestinal and central nervous system irritation, independent of the route of administration, while rapid intravenous administration can lead to convulsions, arrhythmias, severe hypotension, or cardiac arrest (Ma et al., 2016). Most of the adverse reactions of bronchodilators are dose-related. Based on the specific conditions of patients, the rational use of bronchodilators can greatly reduce the incidence and severity of adverse reactions according to the guidelines and experiences. By the way, patients with COVID-19 prescribed inhaled bronchodilators have a risk of contamination through droplet nuclei and aerosols (Cazzola et al., 2021).

While most of these adverse effects of bronchodilators have not been documented in a limited number of clinical studies of COVID-19, attentions need to be paid to them in future investigations. In addition, montelukast (non-bronchodilator) may benefit COVID-19 patients by maintaining the inflammatory/anti-inflammatory balance, preventing respiratory failure through bronchodilator activity, and lowering mortality (Kerget et al., 2022).

## 5 Conclusion and perspectives

Emerging evidences from preliminary clinical results reveal the potential benefits of bronchodilators as repurposed drugs for the treatment of pneumonia or bronchospasm associated with COVID-19. Drug repurposing suggests that attention should be paid to the potential benefits of bronchodilator drugs in the treatment of critically ill patients of COVID-19. It is required to increase the clinical application of bronchodilator drugs, which may bring benefits to critically ill patients. The emerging and reemerging respiratory viral infections will pose new threats to the world in the future. Repurposing use of bronchodilators for COVID-19 would also very likely benefit the therapy for future emerging respiratory infectious disease.
